# Role and Mechanism of Rhizopus Nigrum Polysaccharide EPS1-1 as Pharmaceutical for Therapy of Hepatocellular Carcinoma

**DOI:** 10.3389/fbioe.2020.00509

**Published:** 2020-06-09

**Authors:** Guangyu Chu, Yingying Miao, Kexin Huang, Han Song, Liang Liu

**Affiliations:** ^1^Department of Radiology, The Second Hospital of Jilin University, Changchun, China; ^2^Department of Radiology, China-Japan Union Hospital of Jilin University, Changchun, China; ^3^Department of Histology and Embryology, College of Basic Medicine, Jilin University, Changchun, China

**Keywords:** Rhizopus nigrum polysaccharide, EPS1-1, hepatocellular carcinoma, *in vitro* experiment, nude mice

## Abstract

**Objective:** This work is to study the effect of Rhizopus nigrum polysaccharide EPS1-1 on hepatocellular carcinoma (HCC) *in vitro* and *in vivo*.

**Methods:** HepG2 and Huh-7 cells and nude mice models of liver cancers were used in this study. The cells and nude mice were treated with EPS1-1 at different concentrations. The CCK8 assays were used to measure the proliferation activities of cells, apoptosis was determined with flow cytometry, cell migration was measured by wound-healing assays, cell invasion was evaluated by Transwell assay, and the survival periods of different groups of tumor-bearing mice were compared. Real-time PCR and Western blot were used to measure the expression levels of mRNAs and proteins of the genes related to proliferation, apoptosis, migration, and invasion.

**Results:** *In vitro* experiments revealed that when treated with EPS1-1, HepG2 and Huh-7 cell proliferation activities decreased, while there was an increase for the apoptosis rate, and the migration and invasion capabilities were significantly reduced. *In vivo* experiments showed that EPS1-1 could significantly reduce the tumor growth and lung metastasis of HCC, and prolong the survival periods of tumor-bearing nude mice. Furthermore, EPS1-1 has no apparent damage to the heart, liver, and kidney. Further studies showed that EPS1-1 could affect the expression of proliferation-related genes CCND1 and c-Myc, apoptosis-related genes BAX and Bcl-2, and migration and invasion related genes Vimentin and Slug, thereby affecting the biological process of HCC.

**Conclusion:** EPS1-1 can inhibit the malignant process of HCC *in vitro* and *in vivo*, which indicates that EPS1-1 has the potential value of clinical application as chemotherapy or adjuvant in the treatment of liver cancer.

## Introduction

Hepatocellular carcinoma (HCC) is a common malignant tumor, with its incidence and mortality rate ranks fifth and second in terms of the worldwide cancer death, respectively (Shi et al., 2017; Craig et al., 2019; Sun et al., 2019; Wang Q. et al., 2019). The main reason for the high mortality rate of liver cancer is its high rate of advanced diagnosis. However, at this time, surgical resection is not effective, and there are only a few options available for the treatment of advanced cancer (Vogel and Saborowski, 2020). Therefore, it is an urgent task for researchers to develop drugs that not only have the anti-tumor activity to control the progress of liver cancer but also can be easily used to assist other cancer treatments (Ding et al., 2019; Feng X. et al., 2019; Gao et al., 2019; Ma et al., 2020).

In recent years, there has been increasing attention on the natural antitumor compounds due to their biological activities and little or no side effects (Zhang et al., 2018; Kokudo et al., 2019; Li et al., 2019). Rhizopus nigrum is a zygote filamentous fungus that has been widely used in the brewing and pharmaceutical industries due to its biocatalytic and bio-transformative functions (Pan et al., 2019). Studies have shown that the polysaccharide EPS1-1 of 31930 Da can be extracted from the fermentation liquid of Rhizopus nigrum. The monosaccharide composition of EPS1-1 is rhamnose, xylose, fructose, mannose, dextran, and galactose, with a relative ratio of 16.2:14.4:1:25.8:23.6:48.1 (Massimi et al., 2019). Furthermore, it has been confirmed that EPS1-1 not only significantly inhibits colitis-related colorectal cancer (Bouattour et al., 2019), but also plays a vital role in relieving functional diseases of colorectal cancer mice (Feng B. et al., 2019). In terms of regulating immunity, EPS1-1 can also improve immunity by enhancing cellular and humoral immunity (Hussain et al., 2016). Therefore, as an effective natural antitumor compound, Rhizopus nigrum polysaccharide EPS1-1 has strong potential in the clinical application as an adjuvant medicine.

However, the role of EPS1-1 in HCC has not been studied. Therefore, the study of EPS1-1 in liver cancer has a good innovation and will provide new insights for drug or adjuvant treatment of liver cancer. In this study, we extracted EPS1-1 to study its effects on the proliferation, transformation, migration, and invasion of HCC *in vitro*, and its effect on the survival of tumor-bearing mice *In vivo*. In addition, we also investigated the corresponding mechanisms and evaluated the potential value of EPS1-1 as a chemotherapeutic or adjuvant drug for the clinical treatment of HCC.

## Materials and Methods

### Extraction and Identification of EPS1-1

The Rhizopus nigrum mycelium with better growth was selected for fermentation, decolorization, dialysis, and drying. Then DEAE Sepharose Fast Flow ion-exchange chromatography was used to isolate the crude polysaccharide of Rhizopus nigrum. After that, the polysaccharide EPS1-1 was purified by the dextran gel chromatography column (Pan et al., 2019). Finally, HPLC was used to identify the components within EPS1-1.

### Cell Culture

The HCC cell line, including HepG2 and HuH-7 cells, were purchased from Shanghai Fuheng Biotechnology Co., Ltd. They were cultured in DMEM medium containing 10 % fetal bovine serum, 100 U/mL penicillin, and 100 μg/mL streptomycin. The cells were cultured in a humid environment of 37°C with 5% carbon dioxide in the cell plates. Then, different concentrations of EPS1-1 (25, 50, 100, 200, and 400 μg/mL) were added in the cell plates, and one group of cells without EPS1-1 treatment was used as a control group.

### Cell Proliferation Experiment

Cell proliferation experiments were tested using the CCK8 kit, which was purchased from MedChemExpress, USA. Specifically, cells with a density of 1 × 10^3^ cell/mL were first seeded in 96-well plates and incubated at 37°C for 24 h, EPS1-1 with different concentrations was added. After a certain period (24, 48, and 72 h), the CCK-8 solution (10 μL) was added to each well, and cells were further incubated at 37°C for a while. Finally, the absorbance of samples with different treatments at 450 nM was measured using a spectrophotometer. Three independent experiments were carried out in this test.

### Cell Scratch Test

For the cell scratch test, cells were grown to a 90% confluence in a 6-well culture plate. Then, gently scratch the attached cells with the tip of a sterile 10.0 μL micropipette on the plate surface to form a wound in the monolayer cells, which was defined as the starting time 0 h. After scratching, PBS was used to wash the culture plates three times to remove the detached cells, and after that, the serum-free medium containing different concentrations of EPS1-1 was added to the plates. Cells were then incubated for 24 h in a humid environment at 37°C with 5% carbon dioxide. After incubation for a specific time, cell migration was observed and photographed under a microscope. The relative mobility is represented by the distance at which the scratch decreases, with 0 h as the control. The test was repeated at least three times.

### Cell Invasion Assay

The invasion ability of the cells was evaluated with an 8 μm Transwell Chamber (BD, Bioscience). Specifically, EPS1-1with different concentrations were first added in the cells that were cultured in the cell plates. After 48 h of treatment, cells were trypsinized, resuspended, and subsequently counted in serum-free DMEM medium. 2 × 10^4/^100 μl cells in the serum-free medium were seeded in the upper cavity of Transwell Chamber that was coated with 50 μl matrix gel (BD Biosciences), and DMEM containing 10% FBS was added to the lower cavity. After incubating at 37°C for 12 h, the non-invasive cells in the upper cavity were removed with a cotton swab. Cells moving to the bottom of the membrane were then fixed with a 4% paraformaldehyde. After cell fixation, a crystal violet solution was added to stain the cells for 30 min before a microscope was used to observe the stained cells. The test was repeated at least three times.

### Detection of Cell Apoptosis by Flow Cytometry

To detect the cell apoptosis, the cells were first seeded in a cell plate at 37°C for 24 h, and EPS1-1 was added after incubation. After 48 h, the cells were then collected and stained with Propidium iodide/Annexin V-FITC reagent (BD, Bioscience). Apoptosis was then detected by flow cytometry. In the flow cytometry, Annexin V-FITC emitted green fluorescence, and propidium iodide (PI) emitted red fluorescence. The apoptosis rate was calculated based on the results of flow cytometry. The test was repeated at least three times.

### RNA Extraction and qRT-PCR

Total RNA was extracted from cells using Trizol reagent (Invitrogen), and the purity and concentration of RNA were measured with a spectrophotometer. A PrimeScript RT Reagent Kit kit (Takara) was used for reverse transcription reaction. cDNA obtained by reverse transcription was analyzed by SYBR Green PCR Master Mix (Applied Biosystems) reagent and Roche LightCycler 480 real-time PCR system (Apple BioSosies). The relative expression of mRNA was calculated by the 2^−ΔΔCt^ method, and β-actin was used as an internal reference. The primer sequences are shown in Table 1.

**Table 1:** qRT-PCR primer sequences.

**Genes**	**Primers**	**Sequence (5^**′**^-3^**′**^)**
β-actin	Forward	CATGTACGTTGCTATCCAGGC
	Reverse	CTCCTTAATGTCACGCACGAT
CCND1	Forward	GCTGCGAAGTGGAAACCATC
	Reverse	CCTCCTTCTGCACACATTTGAA
c-Myc	Forward	GGCTCCTGGCAAAAGGTCA
	Reverse	CTGCGTAGTTGTGCTGATGT
BAX	Forward	CCCGAGAGGTCTTTTTCCGAG
	Reverse	CCAGCCCATGATGGTTCTGAT
BCL-2	Forward	GGTGGGGTCATGTGTGTGG
	Reverse	CGGTTCAGGTACTCAGTCATCC
Vimentin	Forward	AGTCCACTGAGTACCGGAGAC
	Reverse	CATTTCACGCATCTGGCGTTC
Slug	Forward	CGAACTGGACACACATACAGTG
	Reverse	CTGAGGATCTCTGGTTGTGGT

### Western Blot

To measure the expression of genes that are related to proliferation, apoptosis, migration, and invasion, Western blot was used to quantify the amount of protein of interest. Specifically, total protein was first extracted from the cells using RIPA protein lysate, which was purchased from Bao Dede Biological, and the total protein was then quantified using the BCA protein quantitative detection kit (Sigma). After protein quantification, 20 μg of protein from each group were taken and separated by 10% sodium lauryl sulfate-polyacrylamide gel electrophoresis (SDS-PAGE). The separated proteins were then electroporated to a polyvinylidene fluoride (PVDF) membrane (Millipore, USA). After that, 5% skim milk powder was used to incubate the membrane at room temperature for 1 h for blocking. After blocking, the primary antibody was added to the sample, and it was then incubated at 4°C overnight. The primary antibody was then recovered afterward, the PVDF membrane was then washed three times with PBST, and the membrane was then incubated with an HRP-labeled secondary antibody, and washed three times with PBST after the incubation. Finally, the ECL chemiluminescence kit (Thermo Fisher Scientific) was used to visualize the protein bands. The antibodies used in this test includes Anti-Cyclin D1 antibody (1:1,000, ab16663, Abcam), Anti-c-Myc antibody (1:1,000, ab32072, Abcam), Anti-Bax antibody (1:1,000, ab32503, Abcam), Anti-Bcl-2 antibody (1:1,000, ab32124, Abcam), Anti-Vimentin antibody (1:1,000, ab8978, Abcam), Anti-SLUG antibody (1:1,000, ab106077, Abcam),Anti-β-actin antibody (1:1,000, ab8226, Abcam). The test was repeated at least three times.

### Animal Experiments and Ethics

Animals used in this research were purchased from Changzhou Cavins Experimental Animal Co., Ltd., and the Animal Experiment Ethics Committee has approved the animal experiments of this research of our institution. Male nude mice of 6–7 weeks old, were cut and open the abdominal cavity and HepG2-GFP cells (1 × 10^6^) suspended in serum-free medium were injected into their livers to construct liver cancer-bearing, mouse models. A total of 24 nude mice were then randomly divided into four groups, and then 100.0 mg/kg, 200 mg/kg, 400 mg/kg EPS1-1 or saline was injected intraperitoneally. At regular intervals, FluorVivo small animal live imaging system was used to detect the green fluorescence of GFP and represents the tumor volume by the size of the fluorescence range. The fluorescence on the lungs of the nude mice represents the positive liver metastasis of liver cancer. At the end of the experiment, all surviving mice were euthanized by cervical dislocation.

### Serum Marker Detection

Serum CK, CK-MB, LDH, ALT, AST, BUN, and Cr detection kits were purchased from Sigma for the serum marker detection. The test was repeated at least three times.

### Statistical Analysis

SPSS (version 22.0) and Graphpad Prism (version 5.0) were used in this study for the statistical analysis. All data in this study were expressed as mean ± standard deviation (mean ± S.D.). The *t*-test and one-way analysis of variance were used to compare the statistical differences between the groups. The difference between different comparisons was considered statistically significant when the *P* < 0.05.

## Results

### Extraction and Identification of EPS1-1

**Figure 1:**
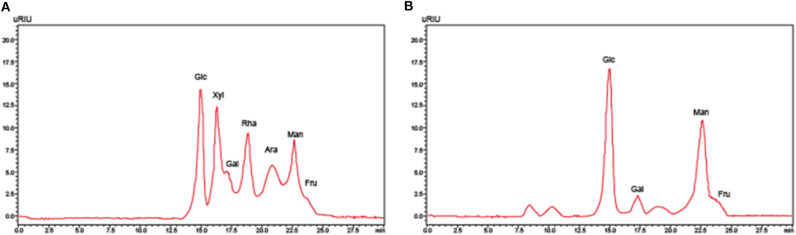
The high-performance liquid chromatography analysis of **(A)** monosaccharide standard sample and **(B)** EPS1-1 hydrolysate.

EPS1-1 was isolated from mycelium of the Rhizopus nigrum mycelium. HPLC was used to identify the components within EPS1-1, which include rhamnose, xylose, fructose, mannose, dextran, and galactose, as shown in Figure 1.

### EPS1-1 Inhibits HCC Proliferation

**Figure 2:**
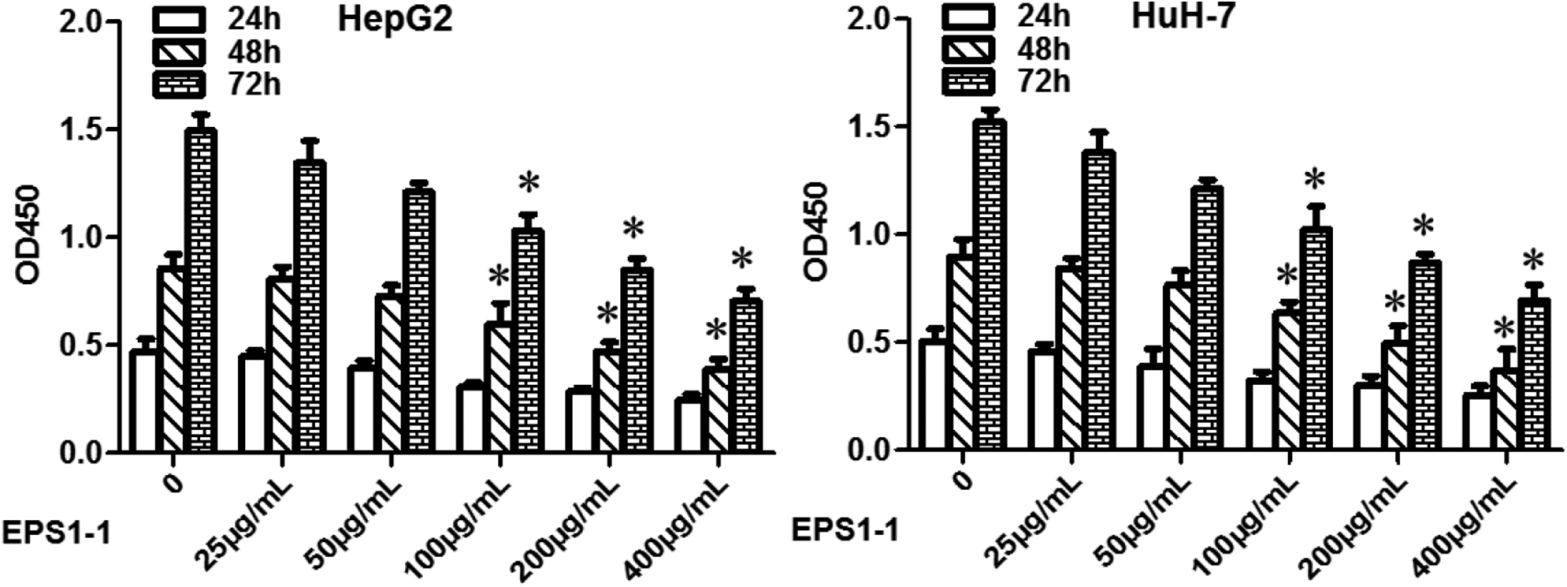
The effect of EPS1-1 on the proliferation activity of HCC detected by CCK8 test. Results are expressed as mean ± standard deviation, *N* = 3, compared with the control group, *P < 0.05.

As shown in Figure 2, after adding 25, 50, 100, 200, and 400 μg/mL of EPS1-1 to HCC cell lines including HepG2 and HuH-7, the cell proliferation ability was decreased significantly (*P* < 0.05) in both HepG2 and HuH-7 when the concentrations of EPS1-1 were at 100, 200, and 400 μg/mL, respectively. The inhibition of EPS1-1 on the proliferation of HCC was in a dose-dependent manner. The results from this test indicate that the EPS1-1 is able to inhibit the HCC proliferation when the EPS1-1 reached a certain level.

### EPS1-1 Promotes Apoptosis of HCC

**Figure 3:**
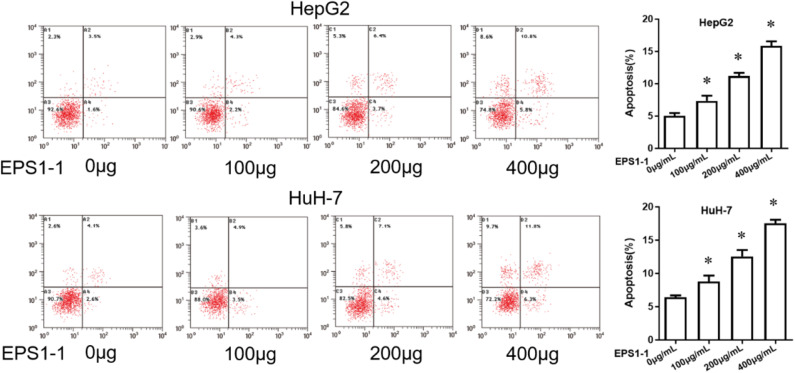
The effect of EPS1-1 on the apoptotic activity of HCC detected by flow cytometry. Results are expressed as mean ± standard deviation, *N* = 3, compared with control group, *P < 0.05.

EPS1-1 was able to inhibit the proliferation of HCC at the concentrations of 100, 200, and 400 μg/mL, as indicated in Figure 2, 100, 200, and 400 μg/mL of EPS1-1 were then added to the HepG2 and HuH-7 to test their effects on the HCC apoptosis. As shown in Figure 3, after adding 100.0, 200.0, and 400.0 μg/mL of EPS1-1 to HepG2 and HuH-7 separately, the apoptosis rates of the cells were then measured by flow cytometry. The results indicated that the apoptosis rates in both HepG2 and HuH-7 were significantly increased (*P* < 0.05), and with a higher level of EPS1-1, a positively correlated increase in the apoptosis rates were observed. The results in this test demonstrated that the addition of the EPS1-1 at ceratin concentration is able to increase the apoptosis rates of HCC.

### EPS1-1 Inhibits Migration of HCC

**Figure 4:**
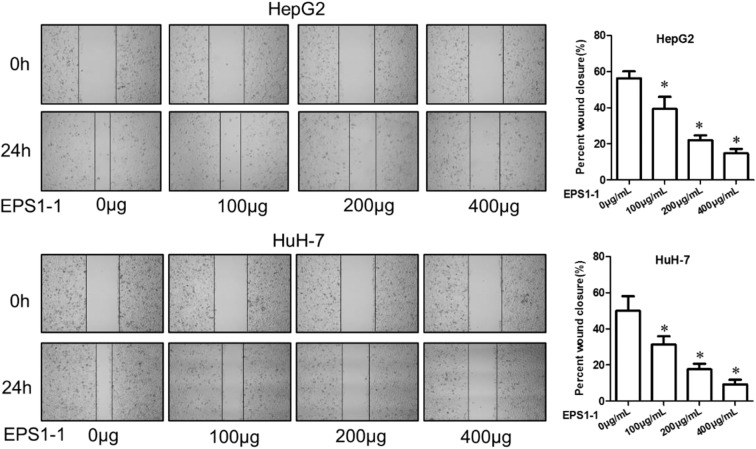
The effect of EPS1-1 on the migration activity of HCC measured by the scratch test. Results are expressed as mean ± standard deviation, *N* = 3, compared with the control group, *P < 0.05.

A cell scratch test was used to measure the effects of the EPS1-1 on the migration of HCC. As shown in Figure 4, after 24 h of adding 100, 200, and 400 μg/mL of EPS1-1 to HepG2 and HuH-7, the percentages of the wound closures were decreased on a concentration-dependent manner, with the 400 μg/mL of the EPS1-1 had the most significant effect on the percentages of wound closure in both HepG2 and HuH-7. Therefore, it appears that the migration ability of HCC was significantly reduced (*P* < 0.05) with the treatment of the EPS1-1.

### EPS1-1 Inhibits Invasion of HCC

**Figure 5:**
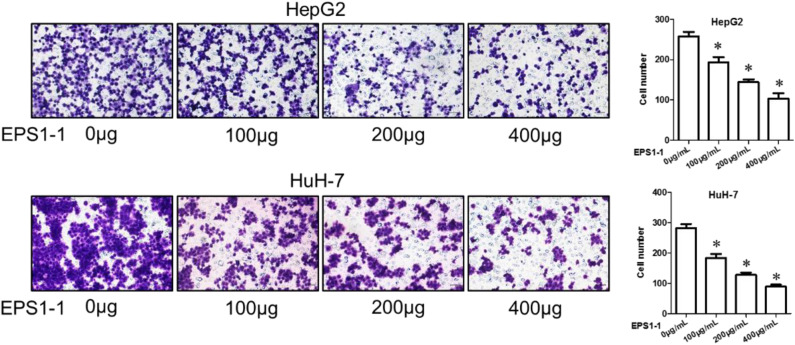
The effect of EPS1-1 on the invasion activity of HCC detected by Transwell tes. Results are expressed as mean ± standard deviation, *N* = 3, compared with the control group, *P < 0.05.

The invasion ability of the HCC was evaluated with an 8 μm Transwell Chamber (BD, Bioscience) as described in the method. As shown in Figure 5, after adding 100.0, 200.0, and 400.0 μg/mL of EPS1-1 to HepG2 and HuH-7 for a certain time, the number of cells that moved to the lower cavity was steadily decreased, and the decrease seems to be negatively correlated with the concentration of the EPS1-1, with the addition of 400 μg/mL of EPS1-1, there were comparatively far fewer cells. In general, the results in this test indicated that the invasion ability of HCC was significantly reduced (*P* < 0.05) with the treatment of the EPS1-1.

### EPS1-1 Affects Tumor Growth, Metastasis, and Survival in Nude Mice Bearing Tumors

**Figure 6:**
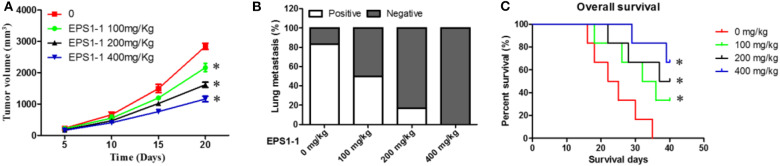
Intraperitoneal injection of EPS1-1 affects tumor growth, metastasis, and survival of tumor-bearing nude mice; **(A)** EPS1-1 inhibits tumor growth; **(B)** EPS1-1 inhibits tumor lung metastasis; **(C)** EPS1-1 promotes survival of nude mice; Results are expressed as mean ± standard deviation, *N* = 3, compared with the control group, *P < 0.05.

To evaluate the effects of the EPS1-1 on the dynamic progression of liver cancer *in vivo*. 100, 200, and 400 mg/kg of EPS1-1 were intraperitoneally injected into the liver tumor-bearing nude mice. The tumor size of tumor-bearing nude mice with different treatments at different time points was then measured. The results showed that compared to the control group, the tumor sizes of the nude mice were significantly reduced in all EPS1-1 treated groups (*P* < 0.05), with the 400 mg/kg of EPS1-1 achieved the most significant tumor size reduction after 20 days of treatment as shown in Figure 6A. The effects of EPS1-1 on the lung metastasis were also determined, as depicted in Figure 6B, where the proportion of positivity was positive correlates with the percentage of the lung metastasis. As the results suggested, with the increasing concentration of EPS1-1 treatment, the percentage of the lung metastasis dropped significantly, with the 400 mg/kg of EPS1-1 treatment, there was almost no observable lung metastasis. To determine the effects of the EPS1-1 on the overall survival of the tumor-bearing nude mice, the overall survival time in each treatment group were also recorded. As suggested in Figure 6C, apparently, all the EPS1-1 treated mice had a significantly longer overall survival time (*P* < 0.05) compared with the control group, and with the mice in the 400 mg/kg of EPS1-1 treatment group achieved the highest percentage of survival rates which was around 60% after 40 days. In contrast, all the mice decreased in the control group after 35 days. Taken all these together, apparently, the treatment of EPS1-1 could reduce the tumor size, mitigate the lung metastasis and increase the overall survival of the tumor-bearing mice, indicating the suppressive effects of EPS1-1 on the tumor *in vivo*.

### EPS1-1 Has a Better Safety

**Figure 7:**
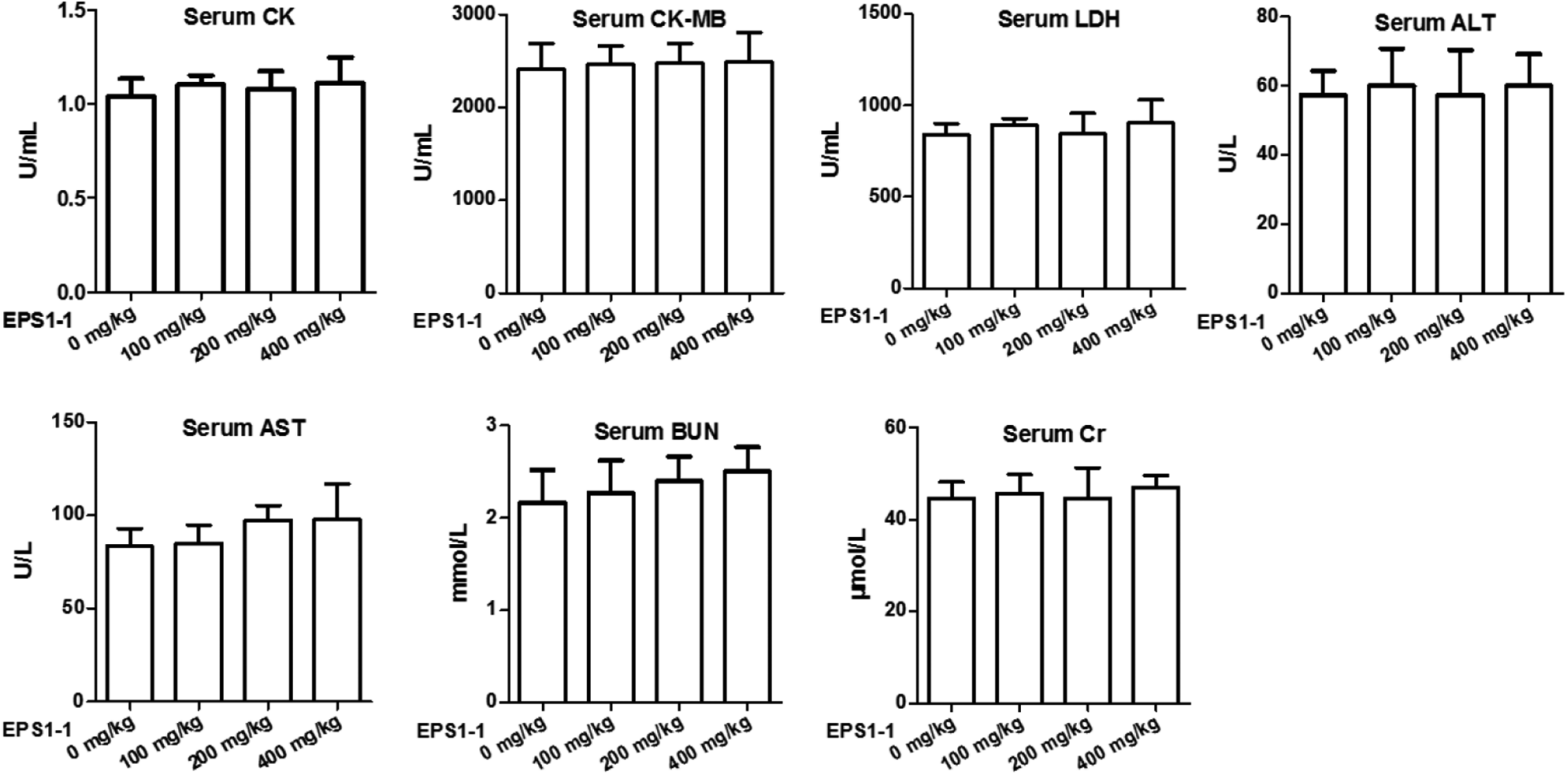
No significant effect on serum CK, CK-MB, LDH, ALT, AST, BUN, and Cr levels in nude mice was observed after EPS1-1 treatment (*P* > 0.05).

To test the safe dosage of the EPS1-1, a variety of serum markers were measured after the EPS1-1 treatment of the nude mice. As shown in Figure 7, after intraperitoneal injection of EPS1-1 at different concentrations (100, 200, and 400 mg/kg), compared with the control group (0 mg/kg), serum CK, CK-MB, LDH, ALT, AST, BUN and Cr levels in the experimental group did not change significantly (*P* > 0.05) suggesting that EPS1-1 has no observable damaging effects to the heart, liver, and kidney in terms of the concentrations of these serum markers. The results in this test indicated that EPS1-1 could be used safely from 100 to 400 mg/kg.

### EPS1-1 Affects Expression of Genes Related to Proliferation, Apoptosis, Migration, and Invasion

**Figure 8:**
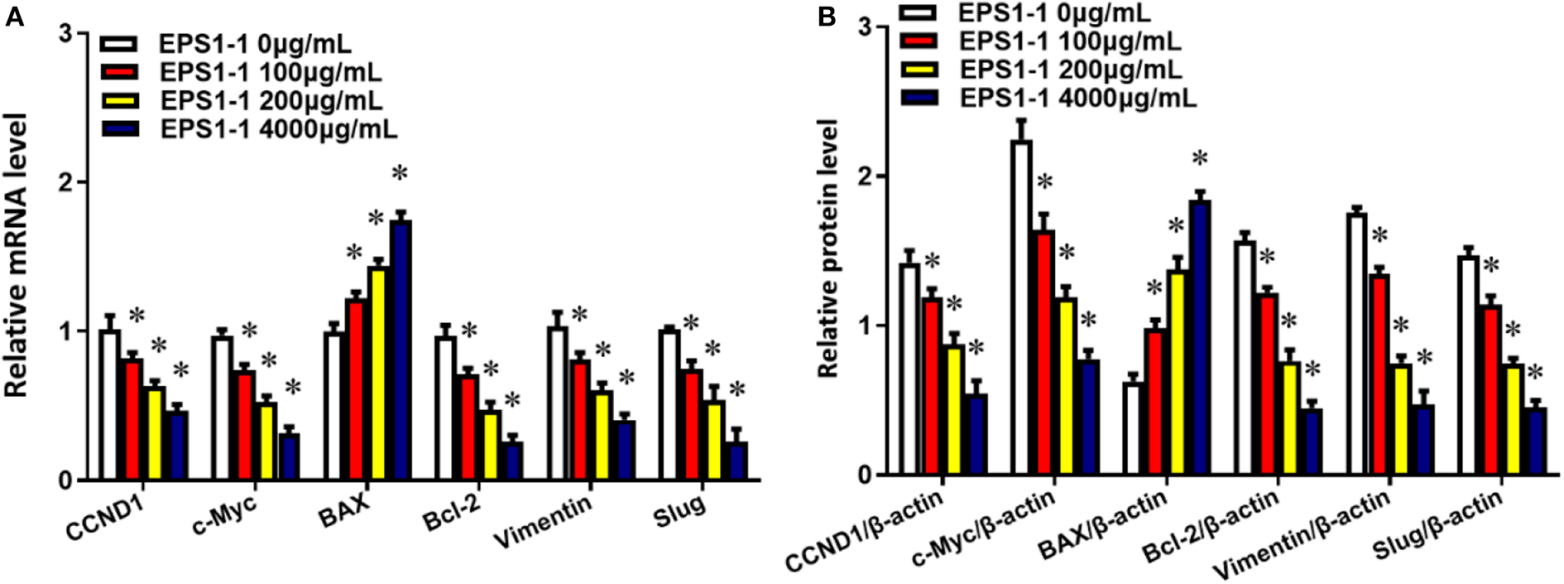
EPS1-1 affects the expression of genes related to proliferation, migration, invasion, and apoptosis. **(A)** mRNA;**(B)** proteins. Results are expressed as mean ± standard deviation, *N* = 3, compared with the control group, *P < 0.05.

To measure the effects of the EPS1-1 on the genes mRNA and proteins that are related to cell proliferation, apoptosis, migration, and invasion, the expressions were determined with the qRT-PCR and Western blot. As shown in Figures 8A,B, after adding 100, 200, and 400 μg/mL of EPS1-1 to HepG2, the expressions of CCND1, c-Myc, BAX, Bcl-2, Vimentin, as well as Slug were measured. The results showed that EPS1-1 could affect the expression of genes mRNA and proteins related to proliferation, apoptosis, migration, and invasion differently. Specifically, the addition of EPS1-1 at 100, 200, and 400 μg/mL were all able to decrease the expression of CCND1, c-Myc, Bcl-2, Vimentin as well as Slug significantly (*P* < 0.05), with the EPS1-1 at 400 μg/mL showed the most significant inhibition. On the other hand, the treatment of EPS1-1 at all tested concentrations was able to increase the expression of BAX protein, and similarly with the EPS1-1 at 400 μg/mL achieved the most significant effect.

## Discussion

Recently, many reports studying polysaccharides of natural origin have indicated that there are a variety of biological functions, such as anti-tumor, immune stimulation, antibacterial, anti-oxidant, with various polysaccharides (Song et al., 2008; Chen et al., 2010, 2019; Opoku-Temeng et al., 2019; Wang X. et al., 2019; Zhang B. et al., 2019). The antitumor effect of these polysaccharides is mainly exerted by regulating the host's immune function. However, the direct inhibition of tumor cells has also conferred some anti-tumor effects of polysaccharides (Chen et al., 2013). An increasing amount of evidence shows that fungal-derived polysaccharides can effectively kill or inhibit cancer cells and are less toxic to healthy cells. These substances cause a variety of changes in cancer cells, such as cellular stress reactions, disruption of the expression of signaling molecules, induction of cell death, cell cycle arrest and autophagy (Huang et al., 2012; Jiang et al., 2019). Therefore, fungal polysaccharides that can induce cancer cell apoptosis are expected to become potential antitumor drugs.

In this study, we extracted polysaccharide EPS1-1 from Rhizopus nigrum and studied its inhibitory effect on HCC *in vitro* and its effect on the survival of tumor-bearing nude mice *in vivo*. *In vitro* experiments show that EPS1-1 at 100, 200, and 400 μg/mL can significantly affect the proliferation activity of HCC, promote its apoptosis, and inhibit its migration and invasion. Further, *in vivo* animal experiments show that intraperitoneal injection of Rhizopus nigrum polysaccharide EPS1-1 can significantly inhibit tumor growth and lung metastasis *in vivo*, and prolong the survival time of tumor-bearing nude mice. Moreover, EPS1-1 has no apparent damaging effects on the heart, liver, and kidney, and has better safety. Therefore, our results indicate that EPS1-1 has significant tumor-suppressive activity in liver cancer. Previous studies on EPS1-1 have shown that EPS1-1 has a significant inhibitory effect on colorectal cancer in animals (Yu et al., 2018a; Song et al., 2019), and EPS1-1 can inhibit tumor invasion and angiogenesis *in vivo* as well as inhibiting tumor metastasis both *in vitro* and *in vivo* (Yu et al., 2018b). Another polysaccharide RPS of Rhizopus nigrum can induce apoptosis and G2/M arrest of gastric cancer BGC-823 cells and play an antitumor role in gastric cancer (Chen et al., 2013). The results in this study demonstrated the antitumor effect of EPS1-1 in liver cancer, which is not only a confirmation of previous research but also an extension of the effect of EPS1-1.

In addition, our further research showed that EPS1-1 regulated the expression of oncogenes and tumor suppressor genes, thereby through which might exert its biological function. These genes include cell proliferation promoting factors including CCND1 and c-Myc (Wang G. et al., 2019; Zhang D. et al., 2019), cell apoptosis regulators BAX and Bcl-2 (Zhang Q. et al., 2019; Hu et al., 2020), and factors that promote cell migration and invasion, such as Vimentin and Slug (Lillo Osuna et al., 2019; Shahid et al., 2019). Study by Song et al. (2019) showed that EPS1-1 could reduce the expression of oncoproteins such as COX-2, β-catenin, CyclinD1, and C-Myc, and down-regulate the expression of proliferation-related proteins including Ki-67 and PCNA and the anti-apoptotic gene Bcl-2, up-regulate the expression of pro-apoptotic genes such as p53 and Bax, reduce the number of CD68, F4/80, and NF-κB positive cells, as well as reducing the concentrations of inflammatory factors in serum including TNF-α and IL-6. This study is a further improvement and supplement of the antitumor mechanism of EPS1-1 and provides a theoretical basis for the antitumor effect of EPS1-1.

## Conclusion

In this study, we found that EPS1-1 can inhibit the malignant process of liver cancer cells *in vitro* by affecting the proliferation, apoptosis, migration, and invasion of HCC. The animal studies demonstrated that EPS1-1 can significantly inhibit tumor growth and lung metastasis *in vivo*, and prolong the survival time of nude mice bearing tumors without significant damage to the heart, liver, and kidneys, and has excellent safety. Results in this study indicate that EPS1-1 has potential value as a chemotherapeutic or adjuvant drug in the clinical application of liver cancer treatment.

## Data Availability Statement

The raw data supporting the conclusions of this article will be made available by the authors, without undue reservation, to any qualified researcher.

## Ethics Statement

The animal study was reviewed and approved by the Animal Care and Use Committee at Jilin University.

## Author Contributions

HS and LL proposed and designed the experiments. GC and YM carried out the experiments with the help of KH and HS. GC and YM drafted the manuscript and interpreted the data. KH, HS, and LL revised the manuscript.

## Conflict of Interest

The authors declare that the research was conducted in the absence of any commercial or financial relationships that could be construed as a potential conflict of interest.
